# The mitochondria-targeted sulfide delivery molecule attenuates drugs-induced gastropathy. Involvement of heme oxygenase pathway.

**DOI:** 10.1016/j.redox.2023.102847

**Published:** 2023-08-12

**Authors:** Katarzyna Magierowska, Dagmara Wójcik-Grzybek, Edyta Korbut, Dominik Bakalarz, Grzegorz Ginter, Aleksandra Danielak, Sławomir Kwiecień, Anna Chmura, Roberta Torregrossa, Matthew Whiteman, Marcin Magierowski

**Affiliations:** a Department of Physiology, Jagiellonian University Medical College, Cracow, Poland; b Department of Forensic Toxicology, Institute of Forensic Research, Cracow, Poland; c University of Exeter Medical School, University of Exeter, Exeter, United Kingdom

**Keywords:** Gastric mucosa, Hydrogen sulfide, Heme oxygenase, Mitochondria, Non-steroidal anti-inflammatory drugs

## Abstract

Hydrogen sulfide (H_2_S) signaling and H_2_S-prodrugs maintain redox balance in gastrointestinal (GI) tract. Predominant effect of any H_2_S-donor is mitochondrial. Non-targeted H_2_S-moieties were shown to decrease the non-steroidal anti-inflammatory drugs (NSAIDs)-induced gastrotoxicity but in high doses. However, direct, controlled delivery of H_2_S to gastric mucosal mitochondria as a molecular target improving NSAIDs-pharmacology remains overlooked.

Thus, we treated Wistar rats, i.g. with vehicle, mitochondria-targeted H_2_S-releasing AP39 (0.004–0.5 mg/kg), AP219 (0.02 mg/kg) as structural control without H_2_S-releasing ability, or AP39 + SnPP (10 mg/kg) as a heme oxygenase (HMOX) inhibitor. Next, animals were administered i.g. with acetylsalicylic acid (ASA, 125 mg/kg) as NSAIDs representative or comparatively with 75% ethanol to induce translational hemorrhagic or necrotic gastric lesions, that were assessed micro-/macroscopically. Activity of mitochondrial complex IV/V, and DNA oxidation were assessed biochemically. Gastric mucosal/serum content of IL-1β, IL-10, TNF-α, TGF-β1/2, ARG1, GST-α, or phosphorylation of mTOR, NF-κB, ERK, Akt, JNK, STAT3/5 were evaluated by microbeads-fluorescent xMAP®-assay; gastric mucosal mRNA level of HMOX-1/2, COX-1/2, SOD-1/2 by real-time PCR.

AP39 (but not AP219) dose-dependently (0.02 and 0.1 mg/kg) diminished NSAID- (and ethanol)-induced gastric lesions and DNA oxidation, restoring mitochondrial complexes activity, ARG1, GST-α protein levels and increasing HMOX-1 and SOD-2 expression. AP39 decreased proteins levels or phosphorylation of gastric mucosal inflammation/oxidation-sensitive markers and restored mTOR phosphorylation. Pharmacological inhibition of HMOX-1 attenuated AP39-gastroprotection.

We showed that mitochondria-targeted H_2_S released from very low i.g. doses of AP39 improved gastric mucosal capacity to cope with NSAIDs-induced mitochondrial dysfunction and redox imbalance, mechanistically requiring the activity of HMOX-1.

## 1 Introduction

Hydrogen sulfide (H_2_S) is an endogenously produced signaling molecule that exerts pleiotropic activity within gastrointestinal (GI) mucosa under physio- and pathological conditions [1,2]. However, targeted mitochondrial delivery of H_2_S was not considered as molecular and pharmacological target counteracting drugs-induced toxicity and alterations in gastric mucosal barrier physiology.

H_2_S-releasing chemicals or prodrugs were shown to prevent gastric mucosa against the damage induced by exposure to acute stress, ischemia/reperfusion or non-steroidal anti-inflammatory drugs (NSAIDs), including acetylsalicylic acid (ASA, known as aspirin) [1,3]. H_2_S generation increasing SG1002 (Sulfagenix Inc., USA) also entered early phases of clinical trials [4]. Sodium thiosulfate (STS), which does not release H_2_S itself, exhibits beneficial effects mimicking those of H_2_S, and is an established FDA-approved drug, including for pediatric patients [5,6]. Importantly, new derivatives of NSAIDs, such as H_2_S-naproxen (ATB-346, Antibes Therapeutics Inc., Canada) or H_2_S-ketoprofen (ATB-352) exerted greater GI safety than the parent NSAID in preclinical or clinical studies [7,8]. In fact, NSAIDs are one of the most commonly used and effective analgesic or anti-inflammatory medications but burdened with significant adverse effects in GI tract, predisposing to peptic ulcer disease development, that is therefore caused not only by the H. pylori infection [9]. These events limit their implementation e.g. in cardiovascular pharmacology or ageing-related weakening of physiological GI barrier [10,11]. Redox imbalance and reactive oxygen species (ROS) excess, along with mitochondrial perturbations were suggested to contribute in pathogenesis of NSAIDs-induced gastric lesions and other type of gastric injuries [[12], [13], [14]]. Nevertheless, the detailed impact of NSAIDs on mitochondrial activity in gastric mucosa remains poorly explored. On the other hand, in spite of the fact that the predominant effect of any H_2_S donor is mitochondrial, mitochondria were not considered as a direct molecular targets for H_2_S-prodrugs in terms of attenuation of NSAIDs gastrotoxicity [15,16].

Several H_2_S-releasing molecules have been identified or developed, including e.g. non-targeted sodium hydrosulfide (NaSH), 4-hydroxythiobenzamide, GYY4137, esterase-sensitive BW-HS platform or mitochondria-targeted AP39 or AP123 molecules [[17], [18], [19], [20], [21], [22]]. Recently *in vitro* studies showed that GYY4137 affected oxygen consumption in endothelial cells due to the modulation of mitochondrial activity [23]. In fact, all donors cause mitochondrial H_2_S to increase but AP39 requires only 100 nM while GYY4137 needs even 400 μM to evoke biological effects. NaSH generates spontaneously high levels of sulfide in uncontrollable manner, with low impact on mitochondria [15,24]. Our studies revealed that 5 mg/kg i.g. of NaSH but only 0.1 mg/kg i.g. of AP39 protected gastric mucosa against oxidative injuries [3].

Next to AP39, new classes of targeted H_2_S donors were developed recently, basically almost all of them based on triphenylphosphonium (TPP) moieties. One of them, by Gilbert and Pluth is based on modular click chemistry approach and an esterase-activated caged thiocarbamates scaffold that enabled subcellular localization of sulfide delivery, such as mitochondria targeting [25]. Additionally, another compound, MitoPerSulf which is penicillamine-based persulfide and rapidly releases H_2_S to mitochondria, was shown to be cardioprotective against ischemia-reperfusion injury [26]. Moreover, mitochondria-targeted H_2_S-delivery platform based on diphenylamino-substituted 1,3-dithiolium-4-olate was proposed [27]. Of note, AP39-derived mitochondrial H_2_S release depends on an anethole dithiolethione (ADTOH) while for AP123 on an hydroxythiobenzamide (HTB) moiety [18,28,29]. Both were shown extensively to exert beneficial biological effects or to modulate fundamental mechanisms *in vitro* and in vivo when applied in very low, even nanomolar and picomolar concentrations [19,30,31]. Importantly, AP39 was reported to modulate mitochondrial proteins persulfidation as a main molecular target for H_2_S [20,32]. Indeed, this approach has been successfully used and numerous studies have shown H_2_S generation from AP39 [[15], [18], [22], [33], [34], [35]], as well as other ADTOH and HTB containing molecules [[36], [37], [38]].

These published observations confirm that H_2_S should be delivered where it is needed - to the mitochondria, or when its needed - e.g. during or after hypoxia or oxidative stress and ischemia-reperfusion injury and oxidation, to evoke the most efficient beneficial effects. From there, we could control inflammation, cell division/fate and all aspects requiring energy and redox balance. This could include new approach on controllable and H_2_S-trigered maintenance of gastric mucosal mitochondrial activity to counteract NSAIDs-induced injuries, that remains overlooked so far. Therefore, we evaluated for the first time if i.g. treatment with mitochondria-targeted, H_2_S-releasing AP39 enhances anti-oxidative capacity of gastric mucosa to eliminate/decrease acetylsalicylic acid-induced gastric damage and comparatively - chemical necrosis induced by i.g. administration of ethanol. We also focused on the possible mechanistic involvement of inflammation-sensitive heme oxygenase-1 (HMOX-1) pathway and the downstream signaling profile accompanying the gastroprotective effect of controlled mitochondria-targeted H_2_S delivery using AP39, including but not limited to mammalian target of rapamycin (mTOR). Importantly, the interplay between mTOR and HMOX-1 was previously suggested in limited number of available publications.

## 2 Materials and methods

### 2.1 In vivo experimental design, examination of gastric lesions, mucosal biopsies collection and storage

This study was based on fifty male Wistar rats (average weight of 220–300 g), fasted for 12-16 h with free access to tap water before each experiment. Protocols and procedures were approved by the 1st Local Ethical Committee for Care and Use of Experimental Animals, held by Faculty of Pharmacy, Jagiellonian University Medical College in Cracow (Decision No.: 311/2019, Date: July 17, 2019; Decision No.: 661/2022, Date: September 27, 2022). Experiments were run following replacement, refinement or reduction (the 3Rs) principle and the ARRIVE guidelines. AP39 and AP219 were synthesized in-house with the purity >95%, as previously described [18,39,40]. Other compounds and chemicals were purchased from Merck (previously Sigma Aldrich, Schnelldorf, Germany).

We used in our study an animal model of aspirin-induced gastric damage that was established and implemented in routine experimental setting (in our laboratory and others) [[41], [42], [43]]. Acetylsalicylic acid (ASA), next to naproxen are the most widely used in daily clinical practice and the most effective NSAIDs, due to the strong anti-inflammatory and analgesic effectiveness. Therefore, we selected the most clinically relevant representative of NSAID such as ASA.

Animals were randomized to experimental groups (5 rats each), pretreated i.g. by orogastric tube with 1 mL of 1) dimethyl sulfoxide (DMSO)/H_2_O (1:9) as vehicle, 2) H_2_S prodrug, [(10-oxo-10-(4-(3-thioxo-3H-1,2-dithiol-5yl)phenoxy)decyl) triphenylphosphonium bromide] (AP39; 0.004–0.5 mg/kg), 3) its mitochondria-targeted analogue, 9-(carboxynonyl)triphenyl-phosphonium (AP219), without the H_2_S-releasing ability as negative control (NC-AP39) [18,39,44,45]. After 30 min, rats were treated i.g. with 1.5 mL of acetylsalicylic acid (ASA (A5376, Merck), 125 mg/kg), following the well-established and validated model of NSAIDs-induced gastric mucosal erosions [43]. AP39 (0.02 mg/kg as a dose capable to reduce the area of ASA-injury by more than 50%) was also co-administered with HMOX-1 inhibitor, tin protoporphyrin IX (SnPP (sc-203452A, Santa Cruz Biotechnology, Dallas, TX, USA), 10 mg/kg i.g.), based on previously implemented protocol [[46], [47], [48], [49]]. Comparatively, separate groups of rats were treated i.g. with vehicle or AP39 (0.02 mg/kg) and after 30 min administered with 1 mL of 75% ethanol to induce necrotic gastric mucosal injuries [50]. Additional control group (Intact) included rats with healthy gastric mucosa without any lesions (not exposed to the treatments with tested compounds).

Blood samples were collected from *vena cava*. Serum was separated and stored at −80 °C until further analysis. The stomach was resected, opened along the greater curvature and pinned out for macroscopic planimetric examination. Gastric erosions area was expressed in mm^2^. Gastric mucosal biopsies were scraped off using a glass slide and frozen in liquid nitrogen.

Gastric tissue segments were also excised and fixed in 10% buffered formalin solution for microscopic analysis by a light microscope (AxioVert A1, Carl Zeiss, Oberkochen, Germany) and ZEN Pro 2.3 software (Carl Zeiss, Oberkochen, Germany). Histological staining was performed using hematoxylin/eosin (H&E) or alcian blue/periodic acid-Schiff/alcian blue (AB/PAS). Our model of ASA-evoked gastric injury allows the investigator to induce gastric mucosal damage with specific morphology allowing to precisely and quantitatively measure the area of the damage in mm^2^ as a primary endpoint. Microscopic appearance is supportive but it is not crucial to define the gastroprotective effects of tested compounds. We supplemented our data evaluating the gastroprotective effect of AP39 applied i.g. in a protective dose of 0.02 mg/kg vs ASA- and comparatively, ethanol-induced gastric mucosal damage at microscopic level. Erosions/necrotic or inflammatory spots were assessed based on our already published protocol [50], using the following scoring criteria/points.

i) 0 - no erosion/necrosis/inflammation, 1 - length of injury <500 μm, 2 - length of injury 501–1000 μm, 3 - length of injury 1001–2000 μm, 4 - length of injury >2000 μm,
ii) 0 - no erosion/necrosis/inflammation, 1 - depth of injury <500 μm per tissue section, 2 - depth of injury >500 μm per slide, 3 - depth of injury—erosion reaching submucosal layer,
iii) 0 – no hemorrhage, 1 – hemorrhage.

Sums of the abovementioned scoring criteria for each injury was calculated and expressed as median with range for each group.

### 2.2 Determination of deoxyguanosine (8-OHdG) level in gastric mucosa

Gastric mucosal content of oxidative DNA damage marker, 8-OHdG was evaluated using ELISA kit (589320), Cayman Chemical, Ann Arbor, MI, USA) following previously described protocol [ [51,52]]. Briefly, total DNA was isolated from gastric mucosa in a single use spin columns using GeneMATRIX Tissue DNA Purification Kit (cat. no. E3550, EURx, Gdansk, Poland). DNA concentration was measured using NanoDrop One Microvolume UV–Vis Spectrophotometer (Thermo Fisher Scientific, Waltham, MA, USA). DNA underwent the digestion using nuclease P1 and further incubation with alkaline phosphatase. Next, DNA samples were mixed with acetylcholinesterase (AChE)-conjugated monoclonal antibodies and were added to the 96-wells plate precoated with the goat polyclonal IgG antibodies, in line with blank, non-specific binding control, maximum binding control and standards. The plate was incubated at 4 °C overnight. The plate was washed and incubated at room temperature for 2 h with Ellman’s Reagent (containing the substrate to AChE). The yellow color product of the enzymatic reaction was measured spectrophotometrically at 412 nm using microplate reader (Tecan Sunrise, Männedorf, Switzerland). Results are expressed as 8-OHdG concentration normalized to 1 μg of total DNA per sample.

### 2.3 Evaluation of complex IV and complex V activity in gastric mucosa

Complex IV activity was assessed using the Complex IV Human Enzyme Activity Microplate Assay Kit (ab109909, Abcam) following the manufacturer's protocol [53,54]. Decrease in the absorbance at 550 nm resulting from the oxidation of reduced cytochrome *c* was determined with a microplate reader (Tecan Sunrise, Männedorf, Switzerland). ATP production was measured in gastric mucosal biopsies using ATP synthase Specific Activity Microplate Assay Kit (ab109716, Abcam), according to the manufacturer’s protocol [55,56]. The enzyme ATP synthase evokes the reversible catalysis of ATP to ADP and phosphate. Thus, ATP synthase activity was measured as the rate of ATP hydrolysis to ADP that is coupled to the oxidation of NADH to NAD+. This was calculated as the rate of change of the absorbance difference at 340 and 405 nm, measured (fluorimetrically) with a microplate reader (Synergy H1, BioTek, Winooski, VT, USA).

### 2.4 Determination of gastric mucosal mRNA fold changes by real-time PCR

Gastric mucosal mRNA expression for HMOX-1, HMOX-2, superoxide dismutase (SOD)1, SOD2, cyclooxygenase (COX)-1, COX-2 was determined by real time PCR as described in details elsewhere [30,57]. Briefly, gastric mucosal RNA was isolated using single use spin columns using GeneMATRIX Universal RNA Purification Kit (EURx, Gdansk, Poland). RNA concentration was measured using NanoDrop One Microvolume UV–Vis Spectrophotometer (Thermo Fisher Scientific, Waltham, MA, USA). Reversed transcription (RT) to cDNA was performed using High-Capacity cDNA Reverse Transcription Kit (MultiScribe™, Applied Biosystems, Life Technologies, Carlsbad, CA, USA). PCR reaction was run in technical triplicates for each sample using the SYBR-green PCR master mix (SG qPCR Master Mix (2x), EURx, Gdansk, Poland) in an Applied Biosystems™ QuantStudio™ 3 PCR System (Thermo Fisher Scientific, Waltham, MA, USA). 4 ng of cDNA for each sample was used per reaction well. HMOX1 expression was determined using forward 5′-GTCCCAGGATTTGTCCGAGG-3′ and reverse 5′ -GGAGGCCATCACCAGCTTAAA-3′ primers. HMOX2 expression was determined using forward 5′ -CCGGGCAGAAAATACCCAGT-3′ and reverse 5′-ATCAGTGCTTCCTTCCGGTG-3′ primers. COX1 expression was determined using forward 5′-AGGTGTACCCACCTTCCGT-3′ and reverse 5′ -CCAGATCGTGGAGAAGAGCA-3′ primers. COX2 expression was determined using forward 5′-ATCAGAACCGCATTGCCTCT-3′ and reverse 5′-GCCAGCAATCTGTCTGGTGA-3′ primers. SOD1 expression was determined using forward 5’-GCGGATGAAGAGAGGCATGTT-3′ and reverse 5’-ACGGCCAATGATGGAATGCT-3′ primers. SOD2 expression was determined using forward 5′-GTGGAGAACCCAAAGGAGAGT-3′ and reverse 5′-GGTCCTGATTAGAGCAGGCG-3′ primers. β-actin (ACTB) and succinate dehydrogenase complex (SDHA) were used as reference genes. ACTB expression was determined using forward 5′-CTAAGGCCAACCGTGAAAAGA-3′ and reverse 5′-TGGTACGACCAGAGGCATAC-3′ primers. SDHA was determined using 5’-TCCTTCCCACTGTGCATTACAA-3’ forward and 5’-CGTACAGACCAGGCACAATCTG-3’ reverse primers. Results obtained for RNA samples isolated from healthy (intact) gastric mucosa and transcribed to cDNA were further used as reference control to normalize the data. Thus, results were analyzed using ΔΔCt method and expressed as mRNA fold change for each gene of interest.

### *2.5* Evaluation of proteins concentration or proteins phosphorylation by xMAP® microbeads fluorescent assays

Proteins of interest were analyzed according to the manufacturer’s protocol and as described elsewhere [30]. Serum concentration of transforming growth factor (TGF)-β1-2, interleukin (IL)-1β, IL-10, tumor necrosis factor (TNF)-α and gastric mucosal concentration of arginase 1 (ARG1), glutathione S-transferase (GST)-α, or phosphorylation of nuclear factor-kappa B (NF-κB), extracellular signal-regulated kinase (ERK), serine–threonine kinase Akt (Akt), signal transducer and activator of transcription (STAT) 3 and 5 was performed using Luminex microbead-based fluorescent assays (Merck Millipore, MA, USA) and Luminex MAGPIX System (Luminex Corp., Austin, TX, USA). Phosphorylation of mTOR was assessed using the same technology (cat no. 48-625MAG, Merck Millipore). In regard to TNF-α, values extrapolation technique was used to predict values outside a data set. Results were normalized to the total protein and/or additionally to the total protein of interest concentration where appropriate.

### 2.6 Statistical analysis

Statistical analysis was performed using GraphPad Prism 9.0 software (GraphPad Software, La Jolla, CA, USA). Results are presented as mean ± SD (N = 5, unless otherwise stated). Data were analyzed by One-way ANOVA with Dunnett’s Multiple Comparison post hoc test or Student’s t-test. For Table 1, median ± range and Mann-Whitney test were implemented. P < 0.05 was considered as statistically significant.

**Table 1:** Microscopic score of gastric mucosal damage induced by i.g. administration of acetylsalicylic acid (ASA, 125 mg/kg) or 75% ethanol (EtOH) in rats treated or not with AP39 (0.02 mg/kg i.g.). Results are median and range of five results per each experimental group. Asterisk (*) indicates significant change as compared with vehicle + ASA (p < 0.05, Mann-Whitney test). Double asterisk (**) indicates significant change as compared with vehicle + EtOH (p < 0.05, Mann-Whitney test).

Experimental group	Microscopic score
Vehicle + ASA	median = 6; min. 5, max. 7
AP39 (0.02 mg/kg) + ASA	median = 4*; min. 3, max. 4
Vehicle + EtOH	median = 6; min. 4, max. 6
AP39 (0.02 mg/kg) + EtOH	median = 0**; min. 0, max. 3

## 3 Results

**Fig. 1:**
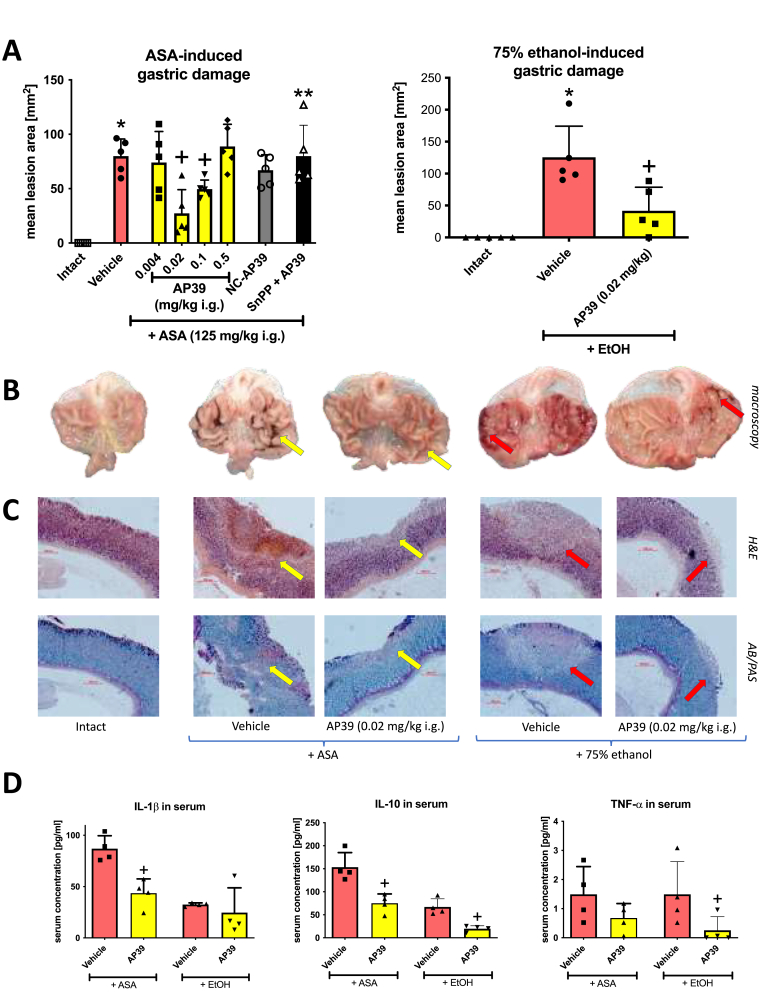
**Gastric mucosal damage induced by i.g. administration of acetylsalicylic acid (ASA, 125 mg/kg) or 75% ethanol (EtOH) in rats treated or not with AP39 (0.004**–**0.5 mg/kg i.g.) or its structural negative control (NC-AP39, 0.02 mg/kg i.g.).** Intact represents healthy gastric mucosa without any lesions (the same for both panels). Results are mean ± SD of five rats per each experimental group. **A:** One group of rats was treated with tin protoporphyrin IX (SnPP, 10 mg/kg i.g.) followed by i.g. administration of AP39 (0.02 mg/kg). Asterisk (*) indicates significant change as compared with intact (*p* < 0.05, ANOVA with Dunnett’s post hoc test). Cross (+) indicates significant change as compared with vehicle (*p* < 0.05, ANOVA with Dunnett’s post hoc test or unpaired *t*-test). Double asterisk (**) indicates significant change as compared with AP39 (0.02 mg/kg i.g.) (*p* < 0.05, unpaired *t*-test). **B, C:** Representative macroscopic images and histological slides of intact gastric mucosa and gastric mucosal damage induced by ASA (yellow arrows) or 75% ethanol (red arrows) in rats pretreated i.g. with vehicle or AP39 (0.02 mg/kg). **C:** Upper panel: hematoxylin/eosin (H&E) stained slides; lower panel: alcian blue/periodic acid-Schiff (AB/PAS) stained slides. **D:** Serum concentration of interleukin (IL)-1β, IL-10, tumor necrosis factor (TNF)-α in rats administered i.g. with acetylsalicylic acid (ASA, 125 mg/kg) or 75% ethanol (EtOH) and treated 30 min earlier with vehicle or AP39 (0.02 mg/kg). Results are mean ± SD of four-five values per each experimental group. Cross (+) indicates significant changes compared with the respective values in vehicle-treated rats (*p* < 0.05, unpaired *t*-test). (For interpretation of the references to color in this figure legend, the reader is referred to the Web version of this article.)

Fig. 1A shows that i.g. treatment with H_2_S-releasing AP39 applied at a dose of 0.02 or 0.1 but not 0.004 or 0.5 mg/kg significantly reduced the area of the damage induced by i.g. administration of acetyl salicylic acid (ASA, 125 mg/kg) (p < 0.05). This data was considered as a primary endpoint that determined the experimental design of further analyses. AP39 applied at a dose of 0.02 mg/kg reduced the ASA-lesions area by more than 50%. Therefore, AP39 at a dose of 0.02 mg/kg was selected for subsequent experiments and analyses. Structural control without ability to release H_2_S (NC-AP39) did not significantly affect the area of ASA-induced damage (Fig. 1A).

AP39 (0.02 mg/kg i.g.) significantly decreased the area of gastric mucosal injury induced by i.g. treatment with 75% ethanol (p < 0.05). Fig. 1B shows macroscopic appearance of healthy (intact) gastric mucosa and the hemorrhagic erosions of gastric mucosa exposed to ASA or 75% ethanol. Treatment with AP39 (0.02 mg/kg i.g.) reduced the area and the range of these injuries. These observations were further confirmed microscopically by H&E and AB/PAS staining where the microscopic score of ASA- or ethanol-induced erosions was significantly decreased in AP39 (0.02 mg/kg i.g.)-treated tissue sections compared to vehicle (Fig. 1C, Table 1).

Treatment with AP39 (0.02 mg/kg i.g.) significantly decreased serum concentration of IL-1β and IL-10 but not TNF-α compared to vehicle in rats administered i.g. with ASA (Fig. 1D). Treatment with AP39 (0.02 mg/kg i.g.) significantly decreased serum concentration of IL-10 and TNF-α but not IL-1β compared to vehicle in rats administered i.g. with EtOH (Fig. 1D). The main of the study was to evaluate gastroprotective effect of AP39 vs ASA-induced gastric damage. Ethanol-induced gastric damage model was implemented only for basic comparison. Therefore, the samples of ethanol-induced gastric damage did not undergo further molecular analyses.

**Fig. 2:**
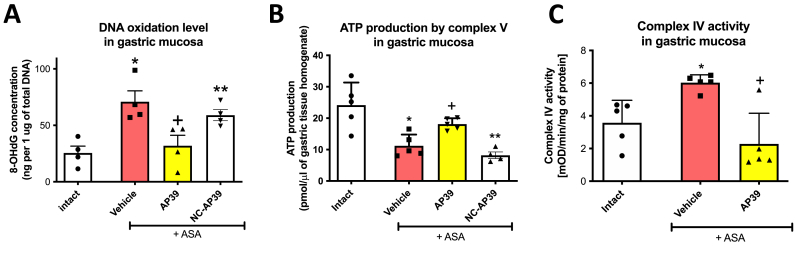
**Concentration of 8-hydroxy-deoxyguanosine (8-OHdG) (A) and the activity of complex V (B) and complex IV (C) in gastric mucosa of rats administered i.g. with acetylsalicylic acid (ASA, 125 mg/kg) and treated 30 min earlier with vehicle, AP39 (0.02 mg/kg i.g.) or NC-AP39 (0.02 mg/kg i.g.).** Intact represents healthy gastric mucosa without any lesions. Results are mean ± SD of four-five samples per each experimental group. Significant changes as compared with the respective values in intact rats are indicated by asterisk (*) (*p* < 0.05, ANOVA with Dunnett’s post hoc test). Cross (+) indicates significant changes as compared with vehicle (*p* < 0.05, ANOVA with Dunnett’s post hoc test or unpaired *t*-test). Significant changes in NC-AP39 group as compared with the respective values in AP39-treated rats are indicated by double asterisk (**) (*p* < 0.05, unpaired *t*-test).

ASA (125 mg/kg i.g.) significantly increased 8-OHdG content in gastric mucosa compared to intact rats (Fig. 2A). However, treatment with AP39 (0.02 mg/kg i.g.) but not NC-AP39 (0.02 mg/kg i.g.) significantly reduced 8-OHdG concentration elevated by ASA compared to vehicle (Fig. 2A). Complex V and complex IV activity in gastric mucosa was significantly enhanced and decreased, respectively, in rats administered with ASA (Fig. 2B and C). Treatment with AP39 (0.02 mg/kg i.g.) significantly elevated and reduced complex V and IV activity, respectively, compared to vehicle (Fig. 2B and C). NC-AP39 (0.02 mg/kg i.g.) did not affect significantly complex V activity compared to vehicle; this parameter was significantly decreased by NC-AP39 (0.02 mg/kg i.g.) vs AP39 (0.02 mg/kg i.g.) (Fig. 2A and B). Thus, based on these results and macroscopic analysis (Fig. 1A), we excluded NC-AP39 from the further biochemical and molecular analyses.

**Fig. 3:**
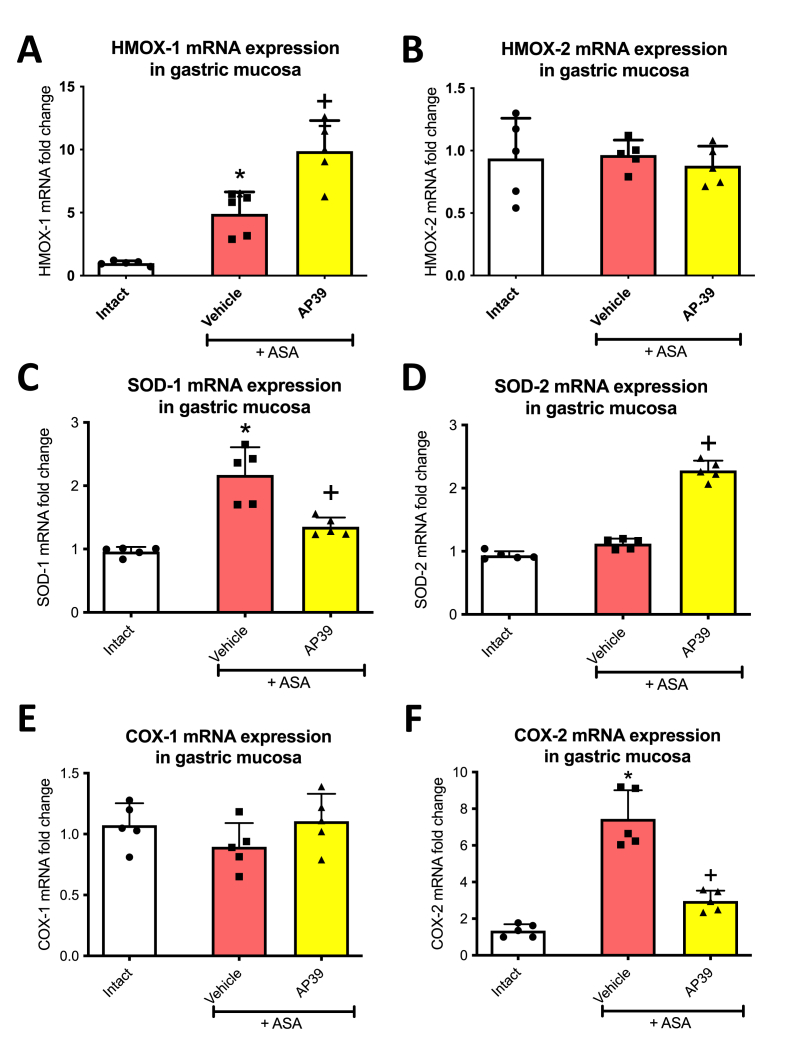
**Gastric mucosal mRNA expression of heme oxygenase (HMOX)-1 (A) and HMOX2 (B), superoxide dismutase (SOD-1) (C), SOD-2 (D), cyclooxygenase 1 (COX-1) (E) and COX-2 (F), in rats administered i.g. with acetylsalicylic acid (ASA, 125 mg/kg) and treated 30 min earlier with vehicle or AP39 (0.02 mg/kg i.g.).** Intact represents healthy gastric mucosa without any lesions. Results are mean ± SD of five rats per each experimental group. Significant changes as compared with the respective values in intact rats are indicated by asterisk (*) (*p* < 0.05, ANOVA with Dunnett’s post hoc test). Cross (+) indicates significant changes as compared with vehicle (*p* < 0.05, ANOVA with Dunnett’s post hoc test).

Fig. 3 shows that ASA (125 mg/kg i.g.) significantly upregulated gastric mucosal mRNA expression of HMOX-1 (Fig. 3A), SOD-1 (Fig. 3C) and COX-2 (Fig. 3F) but not of HMOX-2 (Fig. 3B), SOD-2 (Fig. 3D) or COX-1 (Fig. 3E), compared to intact rats (without any treatment). Treatment with AP39 (0.02 mg/kg i.g.) significantly reduced SOD-1 and COX-2 (Fig. 3C and F), upregulated HMOX-1 (Fig. 3A), SOD-2 (Fig. 3D) and did not affect COX-1(Fig. 3E) or HMOX-2 (Fig. 3B) mRNA expression compared to vehicle.

**Fig. 4:**
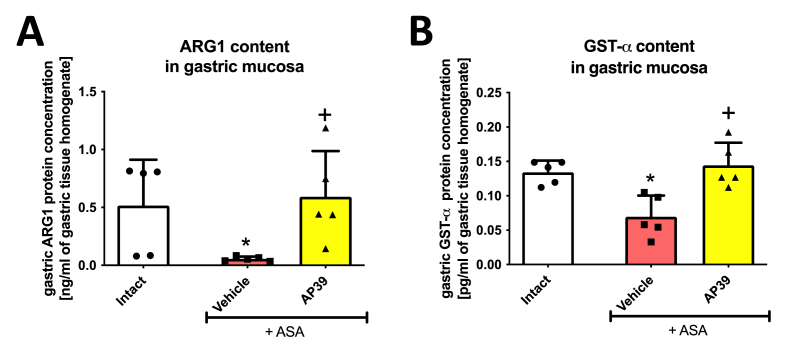
**Protein level ofarginase 1 (ARG1) (A) and glutathione transferase (GST)-α (B) in gastric mucosa of rats administered i.g. with acetylsalicylic acid (ASA, 125 mg/kg) and treated 30 min earlier with vehicle or AP39 (0.02 mg/kg).** Intact represents healthy gastric mucosa without any lesions (B data for intact is derived from the same technical experimental series, published already elsewhere [30]). Results are mean ± SD of five rats per each experimental group. Significant changes compared with intact are indicated by asterisk (*) (*p* < 0.05, ANOVA with Dunnett’s post hoc test or unpaired *t*-test). Cross (+) indicates significant changes compared with vehicle (*p* < 0.05, ANOVA with Dunnett’s post hoc test).

Fig. 4 shows that ASA (125 mg/kg i.g.) significantly decreased gastric mucosal protein level of ARG1 (Fig. 4A) and GST-α (Fig. 4B), compared to intact rats. Treatment with AP39 (0.02 mg/kg i.g.) significantly elevated ARG1 (Fig. 4A) and GST-α (Fig. 4B) compared to vehicle.

**Fig. 5:**
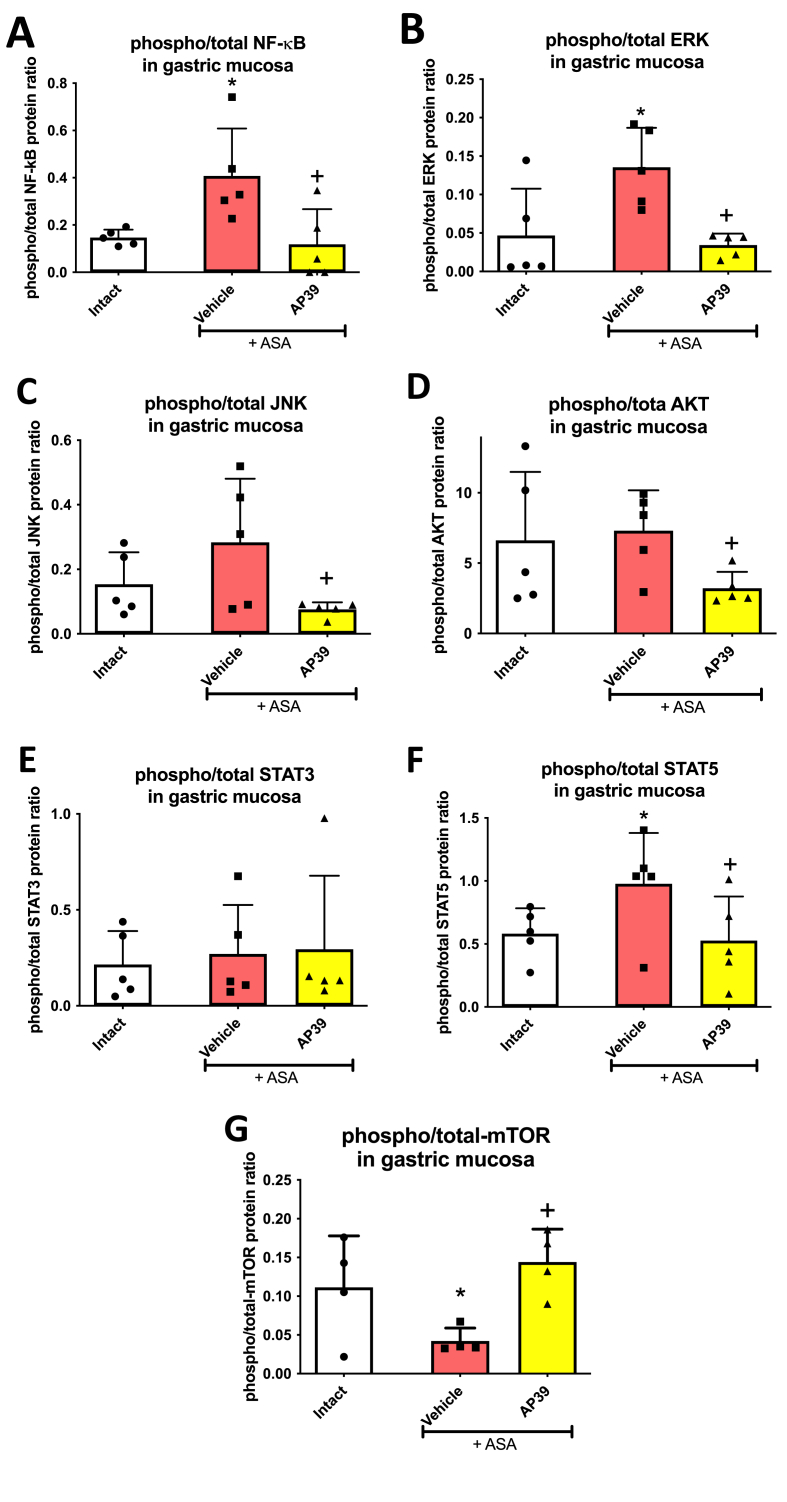
**Gastric mucosal phospho/total protein ratio of nuclear factor-kappa B (NF-κB) (A), extracellular signal-regulated kinase (ERK) (B), c-Jun N-terminal kinases (JNK) (C), serine–threonine kinase Akt (D), signal transducer and activator of transcription (STAT) 3 (E), STAT5 (F) and mammalian target of rapamycin (mTOR) (E) in rats treated i.g. with vehicle or AP39 (0.02 mg/kg) followed by i.g. administration of acetylsalicylic acid (ASA, 125 mg/kg).** Intact represents healthy gastric mucosa without any lesions. Results are mean ± SD of four-five samples per each group. Asterisk (*) indicates significant change compared to intact (*p* < 0.05, ANOVA with Dunnett’s post hoc test or unpaired *t*-test). Cross (+) indicates significant change compared with vehicle (*p* < 0.05, ANOVA with Dunnett’s post hoc test or unpaired *t*-test).

Fig. 5 shows that ASA (125 mg/kg i.g.) significantly increased gastric mucosal phospho/total protein levels ratio of NF-κB (Fig. 5A), ERK (Fig. 5B), STAT5 (Fig. 5F) and decreased phospho/total protein levels ratio of mTOR1 (Fig. 5G) compared to intact rats. Treatment with AP39 (0.02 mg/kg i.g.) significantly decreased gastric mucosal phospho/total protein levels ratio of NF-κB (Fig. 5A), ERK (Fig. 5B), JNK (Fig. 5C), AKT (Fig. 5D), STAT5 (Fig. 5F) compared to vehicle.

**Fig. 6:**
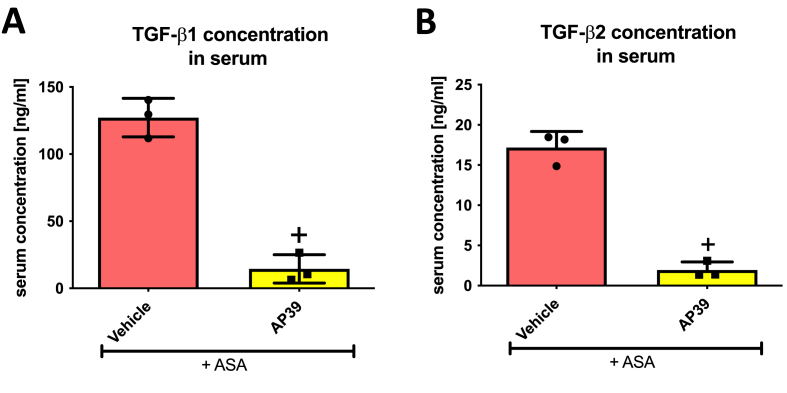
**Serum concentration of transforming growth factor (TGF)-β1 (A) and TGF-β2 (B) in rats administered i.g. with acetylsalicylic acid (ASA, 125 mg/kg) and treated 30 min earlier with vehicle or AP39 (0.02 mg/kg).** Results are mean ± SD of three values per each experimental group. Cross (+) indicates significant changes compared with the respective values in vehicle-treated rats (*p* < 0.05, unpaired *t*-test).

Treatment with AP39 (0.02 mg/kg i.g.) significantly decreased serum concentration of TGF-β1 (Fig. 6A) and TGF-β2 (Fig. 6B) compared to vehicle in rats administered i.g. with ASA.

## 4 Discussion

Gaseous mediators signaling and non-targeted sources of H_2_S were shown to contribute in gastric mucosal integrity and redox balance maintenance [43,50,58,59]. However, we are first to report here that i.g. treatment with mitochondria-targeted H_2_S-prodrug, AP39, dose-dependently eliminated gastric toxicity and redox imbalance induced by acetylsalicylic acid (ASA). ASA is a classic NSAIDs representative, one of the most commonly used in clinical pharmacology. Thus, we selected the well-established and optimized model that was implemented in experimental gastroenterology more than 40 years ago [[41], [42], [43]]. Moreover, in this study we reported comparatively that AP39 attenuated gastric mucosal chemical necrosis induced by 75% ethanol and reduced subsequent systemic inflammatory response. Macro- and microscopic gastroprotection of AP39 was due to its ability to mitochondrial delivery of H_2_S since structural “negative” control did not exert any biological activity. AP39 counteracted ASA-gastrotoxicity when applied in 0.02 and 0.1 mg/kg i.g. but lower or higher doses were not effective. Therefore, the dose of 0.02 mg/kg was taken for comparative experiment on ethanol-induced gastric damage, as well as for molecular and biochemical analyses. We and others confirmed previously the H_2_S-releasing ability of AP39 and that the amount of H_2_S is directly associated to the administered dose of this compound [18,22,29,[33], [34], [35]]. Importantly, our current data showed that treatment with AP39 inhibited NSAID-induced DNA oxidation and restored the activity of mitochondrial complex IV and V in gastric mucosa, all susceptible to i.g. treatment with ASA. However, the targeting molecule without H_2_S-releasing ability (NC-AP39), in contrast to AP39, did not restore DNA oxidation and complex V activity suggesting protection was due to H_2_S. Biological effects of H_2_S rely mainly on persulfidation of proteins [20]. Indeed, H_2_S was described as a direct dose-dependent modulator of mitochondrial complexes activity [16,60]. In our study, we have suggested that the beneficial effects of AP39 were on complex IV and V but this compound also was shown previously to modulate mitochondrial parameters e.g. mitochondrial metabolism or proton leak [29,61]. Altogether, we assume that these fundamental mechanisms, in line with our data support the conclusion that dose-dependent gastroprotection of AP39 vs NSAID damage, observed in this study, is due to the targeted H_2_S delivery that enhances mitochondrial resistance to drugs-induced gastrotoxicity. These observations also correspond with our recent study, where we demonstrated that i.g. treatment with mitochondria-targeted AP39 or RT01 restored anti-oxidative capacity of gastric mucosa and prevented ischemia/reperfusion-induced severe oxidative injuries [30]. Interestingly, sodium thiosulfate (STS) as FDA approved drug for intravenous administration only, is a nontoxic metabolite of H_2_S and could elevate enzymatic and intracellular production of this molecule under oxidative conditions [62]. Mitochondrial enzymes are reported to generate thiosulfate from H_2_S and also to convert thiosulfate to H_2_S [62]. STS exerted e.g. cardioprotective capacity when injected i.p. but unfeasibly high doses were required (e.g. 1 g/kg per day [63,64]) due to its poor availability. This compound was suggested using *ex vivo* experiments to at least maintain mitochondrial functions in isolated rat heart exposed to ischemia-reperfusion, but this still required millimolar concentrations [65]. However, i.p. administration of 1 g/kg of sodium thiosulfate did not impact intestinal and hepatic mitochondrial functions in septic rats [66]. STS was shown to exert cardioprotective effects in mice but when applied orally in drinking water, in a dose of 3 mg/mL per day (that equals approx. 750 mg per day, taking into account the estimated daily intake of 5 mL and the body weight of 20 g [67,68]. FDA approval for STS was based e.g. on clinical trial where this compound required 6 g of i.v. infusion over 5 min [69]. On the other hand, we observed here that H_2_S-releasing AP39 counteracted NSAIDs-induced redox imbalance when applied i.g. at doses several orders of magnitude lower (e.g. 0.02 mg/kg), and in contrast to non-targeted H_2_S-source such as NaSH, that required 5 mg/kg i.g. or esterase-sensitive H_2_S-prodrug BW-HS-101 requiring approx. 15 mg/kg i.g. (50 μmol/kg i.g.), as reported previously [43,50]. This confirms that predominant effect of H_2_S-prodrugs also seems to be mitochondrial in the GI tract, and the low dose of targeted compound (AP39) required to induce gastroprotective effect provides the evidence on huge pharmacological advantage to possible clinical implementation of targeted H_2_S-therapeutic platforms (in GI pharmacology).

Interestingly, we observed here that beneficial effects of AP39 vs ASA gastrotoxicity were in line with enhanced gastric mucosal expression of HMOX-1. HMOX-1 is known as an oxidation- and inflammation-sensitive enzyme exerting cytoprotective activity due to its ability to produce another gaseous mediator, carbon monoxide (CO) [58,70,71]. Indeed, ASA-induced gastric damage was itself followed by upregulated HMOX-1 expression. We noted that pharmacological inhibition of this pathway by SnPP that next to zinc protoporphyrin is widely used as HMOX-1 blocker [72,73], diminished AP39 gastroprotection. This is in parallel with other studies showing that HMOX-1 contributes in the maintenance of GI integrity [74]. Previously, we reported that ASA reduces HMOX-1 activity reflected by the fall in CO content in gastric mucosa [43]. Additionally, we observed that gastroprotection of non-targeted H_2_S donor (NaSH, 5 mg/kg i.g.) against ASA erosions required the co-activity of CO/HMOX-1 pathway but overlooking the detailed mechanism and possible contribution of the mitochondrial integrity [43]. In light of recent finding, it appeared that mitochondrial HO-1 localization has been associated with mitochondrial quality control and cytoprotection [[75], [76], [77]]. Additionally, activation of HMOX-1 by AP39 was reported *in vitro* in human kidney cells [78]. Thus, based on these observations and our current in vivo data, we conclude that pharmacological downstream effect of mitochondrial delivery of H_2_S using AP39 is mechanistically based on the interplay with HMOX-1 that further enhanced beneficial effects of this compound.

Interestingly, it was reported previously in mouse embryonic fibroblasts and H9c2 cardiac myoblasts that HMOX-1 expression was decreased as a result of pharmacological inhibition of mTOR1 pathway [79]. Nevertheless, interplay of mTOR1 and HMOX-1 remains poorly explained. We observed here that the exposure to ASA decreased gastric mucosal phosphorylation of mTOR. Indeed, this pathway was recently shown as a beneficial regulator of gastric epithelial progenitor homeostasis under physiological condition [80]. However, under pathological conditions it can contribute in the development of paligenosis and tumorigenesis [80,81]. Other studies reported that H_2_S could directly interact with mTOR1 protein [82]. On the other hand, it was also demonstrated that anti-depressant effect of H_2_S is due to the activation of mTOR1 signaling [83]. Translation of mitochondrial proteins that are encoded in the nucleus could be regulated by mTOR1. Additionally, mTOR1 was identified in mitochondrial fractions and suggested as a regulator of mitochondrial activity and ATP production [84]. Importantly, we revealed here that AP39-gastroprotection vs. ASA-gastrotoxicity was accompanied by the maintenance of mTOR phosphorylation and ATP production by mitochondrial complex V. Moreover, this is in line with our previous study where we observed that AP39 conserved redox balance in gastric mucosa and counteracted acute oxidative injuries on mTOR1-dependent manner [30]. Altogether, we conclude that AP39-gastroprotection vs. ASA-damage could be due to the direct interaction of delivered H_2_S with complex V supported by the maintained mTOR activity.

This approach is also confirmed by our other results since we proved here that targeted pharmacological modulation of mitochondrial activity by H_2_S released from AP39 was accompanied by the fall in gastric mucosal phosphorylation of inflammation-related and transcription-modulating cellular signaling including NF-kB, ERK, JNK, AKT, STAT5, almost all of them affected by topical administration of ASA. These molecular sensors are known to interact and regulate mTOR and vice versa [84]. Additionally, on systemic level, AP39 decreased the serum level of inflammation-sensitive TGF-β1, TGF-β2, IL-1β, IL-10. These results confirmed the anti-inflammatory properties of AP39 and showed that H_2_S-mediated maintenance of gastric mucosal mitochondrial activity inhibits inflammatory response to chemical gastrotoxicity of the selected, widely used in clinical practice anti-inflammatory drug.

As mentioned above, mTOR1 regulates the expression of mitochondrial proteins encoded in nucleus [84]. Our data shows that targeted activity of H_2_S released from AP39 was reflected by upregulation of mitochondrial SOD-2 expression in contrast to decreased levels of cytoplasmic SOD-1 isoform. This observation reveals that H_2_S-mediated anti-oxidative response of gastric mucosa to NSAIDs damage relies mainly on these organelles functioning. As additional downstream effect of H_2_S-mediated maintenance of the mitochondrial balance in gastric mucosa, restored levels of GST-α and ARG1 were observed. Both markers were decreased by ASA. GST-α contributes in cellular protection and was described as an early marker of intestinal damage [85]. Restoration of GST-α level by AP39 confirmed our previous observation on ischemia/reperfusion-induced gastric injury [30]. ARG1 was shown to be upregulated in gastric mucosa of patients with inactive gastritis [86]. However, increased activity of ARG1 decreases l-arginine level. Furthermore, nitric oxide (NO) content depends on the activity of ARG1 [87,88]. Therefore, our results correspond with Takeuchi et al. who showed the elevated release of NO due to the l-arginine metabolism in gastric mucosa exposed to ASA [89].

It was reported previously by us and others that ASA-induced gastric damage is accompanied by the generation of ROS that led to lipid peroxidation [90,91]. Our studies revealed that ASA-triggered oxidation in gastric mucosa caused DNA oxidation [50]. On the other hand, AP39 was proved to decrease cellular ROS levels in animal model of liver injury and to reduce mitochondrial ROS [29,61,92]. In this study, based on the evaluation of 8-OHG/8-OHdG concentration in gastric mucosa [30,45,50], we observed that ASA-evoked DNA oxidation was decreased in gastric mucosa treated with AP39. This result confirms that mitochondria-dependent AP39 gastroprotection is due to its anti-oxidative properties.

Finally, we would like to note that AP39 prevented gastric mucosa against deep penetration of ASA-induced gastric damage as revealed by our microscopic analysis. Gastric mucosal biopsies (collected carefully without submucosal segments) underwent molecular and biochemical analyses to provide above described data. Therefore, we propose that AP39 directly enhanced epithelial cells defensive capacity but possible impact on the other cells within gastric mucosa could not be definitely excluded.

**Fig. 7:**
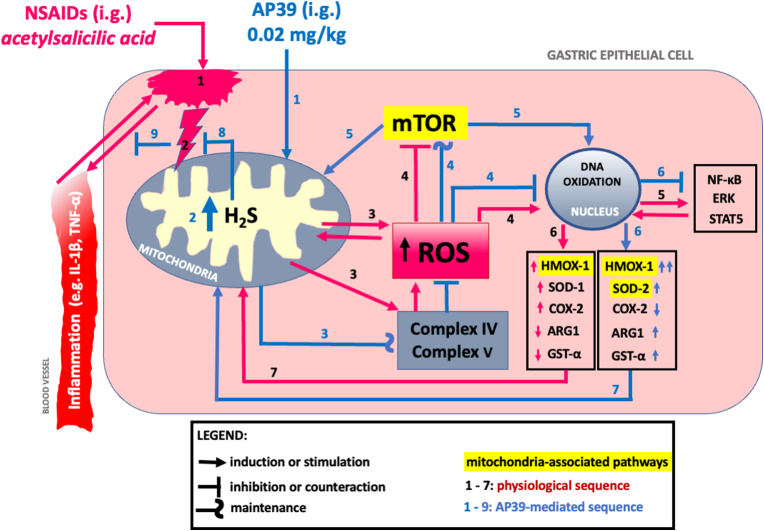
**Overview on the possible sequence of events for NSAIDs-induced gastrotoxicity (red lines) and for the mechanistic approach of mitochondria-targeted AP39 gastroprotection vs. acetylsalicylic acid (ASA) (blue lines).** Abbreviations: NSAIDs: non-steroidal anti-inflammatory drugs; H_2_S: hydrogen sulfide; mTOR: mammalian target of rapamycin; ROS: reactive oxygen species; SOD: sodium dismutase; COX: cyclooxygenase; HMOX: heme oxygenase; ARG: arginase; GST: glutathione S-transferase; ERK: extracellular signal-regulated kinase; STAT: signal transducer and activator of transcription; NF-κB: nuclear factor kappa-light-chain-enhancer of activated B cells; IL: interleukin; TNF: tumor necrosis factor. **Comment:** H_2_S-releasing AP39 increased mitochondrial resistance in gastric mucosa and therefore led to the more effective counteraction to ASA-evoked i) redox imbalance (reflected by altered DNA oxidation and altered complex IV/V activities) and ii) decreased activity of mTOR (that physiologically regulates gastric epithelial progenitor homeostasis). As a downstream effect, AP39 enhanced anti-oxidative cellular response by overexpression of cyto- and mito-protective HMOX-1 and SOD-2. This interplay further stimulated anti-oxidative defensive response supported by mTOR1 homeostasis maintenance. Therefore, ASA-triggered DNA oxidation (by ROS) was blocked, epithelial damage was decreased what prevented the activation/inhibition of subsequent cascade, including the phosphorylation of particular elements of intracellular signaling and systemic inflammatory response. Alterations of SOD-2 were evaluated on mRNA level. Due to the implementation of the selective inhibitor (in line with the gastric mucosal expression analysis), we assumed that AP39-gastroprotection vs. ASA involves the activity of HMOX-1 pathway. (For interpretation of the references to color in this figure legend, the reader is referred to the Web version of this article.)

Taken together, targeted and controlled mitochondrial delivery of H_2_S enhances the ability of these organelles, and in consequence the whole gastric mucosa to counteract the redox imbalance and the hemorrhagic ulcerogenic erosions development, considered as a the main adverse effects of i.g. treatment with NSAIDs. Increased mitochondrial H_2_S-bioavailability due to i.g. administration of AP39 prevented ASA-induced i) DNA oxidation, ii) altered mitochondrial complex IV and V functioning, iii) the fall of anti-oxidative ARG1 and GST-α expression. Moreover, AP39 enhanced the expression of reactive oxygen species scavenging mitochondrial SOD-2. Importantly, pharmacological effectiveness of mitochondria-targeted H_2_S-delivery depends, at least in gastric mucosa, on the activity of anti-oxidative HMOX-1 pathway. As a downstream effect of AP39-derived H_2_S, we reported decreased resolution of inflammation on systemic level and directly in gastric mucosa, reflected by the fall in phosphorylation of cellular signaling regulatory proteins. Possible mechanistic approach is schematically presented and overiviewed on Fig. 7. Our study provides the evidence-based rationale and opens the way for the evaluation of therapeutic properties of targeted H_2_S-prodrugs versus pre-existing GI pathologies, including but not limiting to peptic ulcer disease. We postulate that further implementation of mitochondria-targeted H_2_S-based prodrugs that are effective in very low doses, applied itself or chemically combined e.g. with NSAIDs, could significantly impact the development of gastrointestinal pharmacology. As a “tool” compound, this study has highlighted NSAID-induced gastric injury as a mito-H_2_S “druggable” condition.

## Authors contributions

Conceptualization: K.M., M.M.; Investigation/Experiments: K.M., D.W.G., E.K., D.B., G.G., A.D., S·K., A.C., M.M.; Methodology: K.M., M.M.; Methodology (chemical synthesis): R.T., M.W; Supervision: K.M., M.M.; Funding acquisition: K.M., M.M.; Resources: M.W., M.M.; Visualization: K.M., M.M.; Writing—original draft and revision: K.M., M.M.; Writing—review & editing: K.M., M.W., M.M.; All authors have read and agreed to this version of the manuscript.

## Funding

This study was supported by statutory grants for K.M. (N41/DBS/000877, N41/DBS/000578) and for M.M. (N41/DBS/001140, N41/DBS/000106, N41/DBS/000784) received from 10.13039/100009045Jagiellonian University Medical College (Poland). K.M. was awarded in 2019 by a Fellowship for Outstanding Developing Investigators from Ministry of Science and Higher Education (Poland). Publication was funded (part of the laboratory equipment) by 10.13039/501100007088the Priority Research Area qLife under the program “Excellence Initiative – Research University” at the Jagiellonian University in Krakow (Poland).

## Declaration of competing interest

These authors declare the following financial interests/personal relationships which may be considered as potential competing interests: Matthew Whiteman reports a relationship with MitoRx Therapeutics that includes: equity or stocks. Roberta Torregrossa reports a relationship with MitoRx Therapeutics that includes: employment. Matthew Whiteman has patents awarded and pending for the use of sulfide-delivery molecules.

## Data Availability

Data will be made available on request.
